# Effects on body weight, eating behavior, and quality of life of a low‐energy diet combined with behavioral group treatment of persons with class II or III obesity: A 2‐year pilot study

**DOI:** 10.1002/osp4.464

**Published:** 2020-10-28

**Authors:** Jan Karlsson, Marije Galavazi, Stefan Jansson, Johan Jendle

**Affiliations:** ^1^ University Health Care Research Center Faculty of Medicine and Health Örebro University Örebro Sweden; ^2^ School of Medical Sciences Faculty of Medicine and Health Örebro University Örebro Sweden

**Keywords:** behavior therapy, eating behavior, low‐energy diet, quality of life, weight loss

## Abstract

**Objective:**

Obesity is associated with reduced health‐related quality of life (HRQoL). Outcomes of nonsurgical weight loss treatment on HRQoL are inconsistent and it is unclear how much weight reduction, or what type of treatment, is required for significant improvements. This study aimed to evaluate the effects of a lifestyle intervention program on weight, eating behaviors, and HRQoL, and to describe participants' experiences of treatment.

**Methods:**

This 2‐year intervention trial in persons with class II or III obesity comprised a 3‐month liquid low‐energy diet (880 kcal/d) followed by a 3‐month reintroduction to regular foods, combined with behavioral group treatment.

**Results:**

Fifty‐five participants (73% women) were included, mean (SD) age 43.2 (12.4) years, and mean body mass index 42.0 (6.0) kg/m^2^. Mean weight loss at 6, 12, and 24 months was 18.9%, 13.7%, and 7.2%, respectively. Short‐ and long‐term effects on eating behavior were favorable. Twelve of 14 HRQoL domains were improved at 6 months, compared to eight domains at 12 months. After 24 months, 2 of 14 domains, physical and psychosocial functioning, were improved. The treatment program was well accepted by the participants.

**Conclusions:**

Substantial weight loss after 6 months was associated with extensive improvements in HRQoL, comprising the physical, psychosocial, and mental domains. Significant weight regain was observed between 6 and 24 months follow‐up. Modest weight loss after 24 months was associated with moderate improvement in physical functioning and large improvement in psychosocial functioning. The effect on psychosocial functioning is most likely related to both weight loss and behavioral treatment.

## 1 INTRODUCTION

Obesity is associated with reduced health‐related quality of life (HRQoL), especially among women and those with class III obesity (body mass index [BMI] ≥40 kg/m^2^).^1^ Decreased HRQoL is a common reason why persons with obesity seek treatment for their condition. Improvements in physical and obesity‐specific HRQoL have been demonstrated after bariatric surgery,^2^ while the effects on mental HRQoL are small,^3^ especially in the long term.^1^ Effects on HRQoL of nonsurgical treatment are inconsistent and improvements are mostly of unclear clinical significance.^4^, ^5^ A weight loss of 5%–10% is considered to decrease or eliminate several risk factors and comorbidities associated with obesity,^6^ but may be insufficient to improve most HRQoL domains.^7^ Some studies show that improvements in HRQoL are primarily an effect of the amount of weight loss, but it is uncertain whether other factors also play a role. Therefore, more research is needed to identify which HRQoL domains can be improved and how much weight loss, or what type of treatment, is required for clinically significant improvements.

As obesity is a complex disease, a multidisciplinary approach is required to prevent and treat it. Bariatric surgery is the most effective treatment for achieving long‐term weight loss and improvements of comorbidities.^6^, ^8^ However, surgery is not an option for all people with obesity and only a small proportion of eligible persons undergo surgery,^8^ indicating that other treatment options should be offered.^9^

Very low‐energy diets (VLEDs) of <800 kcal/d consist of meal replacements that can be used for a limited time to achieve rapid weight loss.^10^, ^11^ A 3‐month VLED leads to about 15%–20% weight loss^12^, ^13^ and large initial weight reduction is a strong predictor of long‐term outcome.^14^, ^15^ As with all nonsurgical weight loss treatments, sustainability is a challenge and weight regain after a VLED is common. However, structured and prolonged reintroduction of ordinary food after the VLED phase improves weight loss maintenance after 1 year.^12^ In addition, VLED in combination with behavioral treatment results in greater long‐term weight loss than achieved by monotherapy.^10^ Intensive, multicomponent behavioral interventions are recommended for treating individuals with obesity.^16^ The effect of combining VLED with group‐based behavioral treatment, in a large sample of persons with obesity (*n* = 5965), showed a mean weight loss of 17% in completers (66%) after 1 year.^17^ Most previous studies have used VLED, but liquid‐based low‐energy diets (LEDs) of about 800–900 kcal/d tend to give fewer side effects and can provide equivalent long‐term weight loss.^18^

In the present study, a 2‐year weight loss program was evaluated, which included an initial 3‐month liquid‐based LED (880 kcal/d) followed by 3 months of gradual reintroduction of regular foods, in combination with intensive, multicomponent group‐based behavioral treatment. The aim of this study was to evaluate changes in body weight, eating behavior, and HRQoL, and to describe the participants' experiences of the treatment program.

## 2 MATERIALS AND METHODS

### 2.1 Study design

The study design was a prospective intervention trial. Patients referred to the Obesity Unit, Department of Endocrinology, Örebro University Hospital, Region Örebro County, were considered for inclusion in the study. Inclusion criteria were: age ≥18 years and obesity class II (BMI 35.0–39.9 kg/m^2^) or III (BMI ≥40 kg/m^2^). Exclusion criteria were: eating disorders, current abuse of alcohol or narcotics, heart failure (New York Heart Association 3–4), chronic obstructive pulmonary disease (forced expiratory volume in 1 s ≤50%), liver failure (liver enzymes more than three times the normal level), pregnancy or breastfeeding, type 1 diabetes mellitus, and serious psychiatric disorders. Eligibility to participate in the study was evaluated through a self‐reported questionnaire on current health issues, review of the medical record, meeting with the staff at the Obesity Unit, and at an individual meeting with a physician. The study was approved by the regional ethical review board of Uppsala (Dnr 2011‐379). All participants signed informed consent before inclusion in the study.

### 2.2 Intervention

Participants were instructed to follow a strictly liquid LED (880 kcal/d) for 3 months. Thereafter, the LED was gradually phased out in 3 steps over 3 months; one liquid meal replacement per day was removed every 4 weeks and replaced with an energy‐reduced, regular meal. After 6 months, an individualized, energy‐reduced diet of 1400–1600 kcal/d was recommended.

During the first year, the behavioral program included group sessions (2.5 h) every other week and a total of five individual visits to either (or a combination of) a dietician, physician, and/or physiotherapist. Nine group sessions were offered during the second year. The main and recurring intervention themes discussed during group sessions included self‐monitoring, goal setting, relapse prevention, reducing weight stigma, cognitive restructuring, stimulus control, problem solving, nutrition, eating behavior, physical activity, sedentary behaviors, sleep, and stress management. The group sessions were led by two to three health providers at the Obesity Unit (psychologist, dietitian, physiotherapist, or physician). One of the authors (Marije Galavazi) was involved in the treatment as group leader for the fourth group during the second treatment year. The fee for each group session was 100 Swedish kronor (approximately $11/€10). A high‐cost threshold of 1800 Swedish kronor (approximately $164/€180) was used, which included all visits to outpatient health care per patient and year.

### 2.3 Measures

Body weight, with participants wearing light clothing but no shoes, was measured at the treatment visits by a treatment provider, at baseline (Day 1 of LED treatment) and at 3, 6, 12, and 24‐month follow‐up to the nearest 0.1 kg using electronic scales. Height was measured to the nearest 0.01 m at baseline, with participants in a standing position and without shoes. Body mass index was calculated.

### 2.4 Patient‐reported outcomes

Patient‐reported outcome measures were assessed at baseline and at 6, 12, and 24 months' follow‐up. The questionnaires were sent home to the participants together with a prepaid response envelope.

### 2.5 Eating behavior

The Three‐Factor Eating Questionnaire‐Revised 21‐item measures three eating behaviors: cognitive restraint (conscious restriction of food intake to control body weight or body shape), uncontrolled eating (inability to control eating when feeling hungry or exposed to food), and emotional eating (overeating in response to negative mood).^19^ Scale scores range from 0 to 100 and a higher score indicates higher levels of the respective eating behaviors.

### 2.6 Health‐related quality of life

The Short Form 36‐item Health Survey (SF‐36) measures generic HRQoL and comprises eight domains: physical functioning, role physical, bodily pain, general health, vitality, social functioning, role emotional, and mental health.^20^ Scores range from 0 to 100 and higher scores indicate better HRQoL. The SF‐36 health profile in the study group was compared to a sex‐ and age‐matched general population sample randomly selected from the Swedish SF‐36 normative database (*n* = 8930).^20^ The reference sample comprised 715 persons (72.7% females) with a mean (standard deviation [SD]) age of 43.2 (12.4) years.

### 2.7 Obesity‐specific HRQoL

The Obesity‐related Problems (OP) scale is an obesity‐specific HRQoL instrument developed for measuring the impact of obesity on psychosocial functioning in two domains: distress and avoidance.^21^, ^22^ Subjects are asked to rate how bothered they are by their obesity in different social situations and to what extent they avoid such situations. Scores range from 0 to 100, and higher scores indicate dysfunction. A distress score <40 is interpreted as mild, 40–59 as moderate, and ≥60 as severe dysfunction.^21^

### 2.8 Domain‐specific HRQoL

The Hospital Anxiety and Depression (HAD) scale measures two domains: symptoms of anxiety and depression.^23^ Scores range from 0 to 21 and higher scores represent more symptoms. Individual scores are classified as follows: <8 = normal range, 8–10 = possible mood disorder, and ≥11 = probable mood disorder.

The Brief Pain Inventory‐Short Form (BPI‐SF) measures pain severity and interference.^24^ The severity scale includes ratings of worst, least, average, and current pain intensity. The interference scale measures how much the pain has disturbed daily life in seven areas: general activity, walking, work, mood, enjoyment of life, relations with others, and sleep. Scores range from 0 to 10 and higher scores indicate higher levels of pain severity and interference. Cutoff values for pain severity have been suggested to be: 0 = no pain, 1–3 = mild, 4–6 = moderate, and 7–10 = severe pain.^25^

### 2.9 Patient‐reported experience measures

Study‐specific patient‐reported experience measure questionnaires after 3, 6, and 12 months were used to evaluate participants' experiences of the treatment program, that is, difficulties in following the LED, gastrointestinal side effects and fatigue during the LED, motivation to continue treatment, and satisfaction with social support.

### 2.10 Statistical analysis

The Mann–Whitney *U*‐test was used to test differences in SF‐36 scale scores between the study sample and a sex‐ and age‐matched reference sample from the Swedish SF‐36 normative database. Within‐group change was tested with paired samples *t*‐test or Wilcoxon signed ranks test. The effect size of a between‐group difference was estimated by calculating Cohen's *d*, that is, the mean difference between groups, divided by the pooled SD. Effect size of within‐group change was estimated by calculating the standardized response mean (SRM), that is, the mean change divided by the SD of change. Effect size/SRM was evaluated according to standard criteria: <0.20 = trivial, 0.20–0.49 = small, 0.50–0.79 = moderate, and ≥0.80 = large.^26^ Correlations between variables were tested using Pearson's correlation coefficient.

All *p*‐values were two‐tailed and *p* < 0.05 was considered statistically significant. Analysis was performed using SAS, version 9.4 (SAS Institute Inc.).

## 3 RESULTS

### 3.1 Participants' characteristics at baseline

Four groups consisting of a total of 55 participants (73% women) started weight loss treatment. The mean age (SD) was 43.2 (12.4) years (range 19–72) and the mean BMI was 42.0 (6.0) kg/m^2^ (range 35–60).

### 3.2 Weight change

**FIGURE 1:**
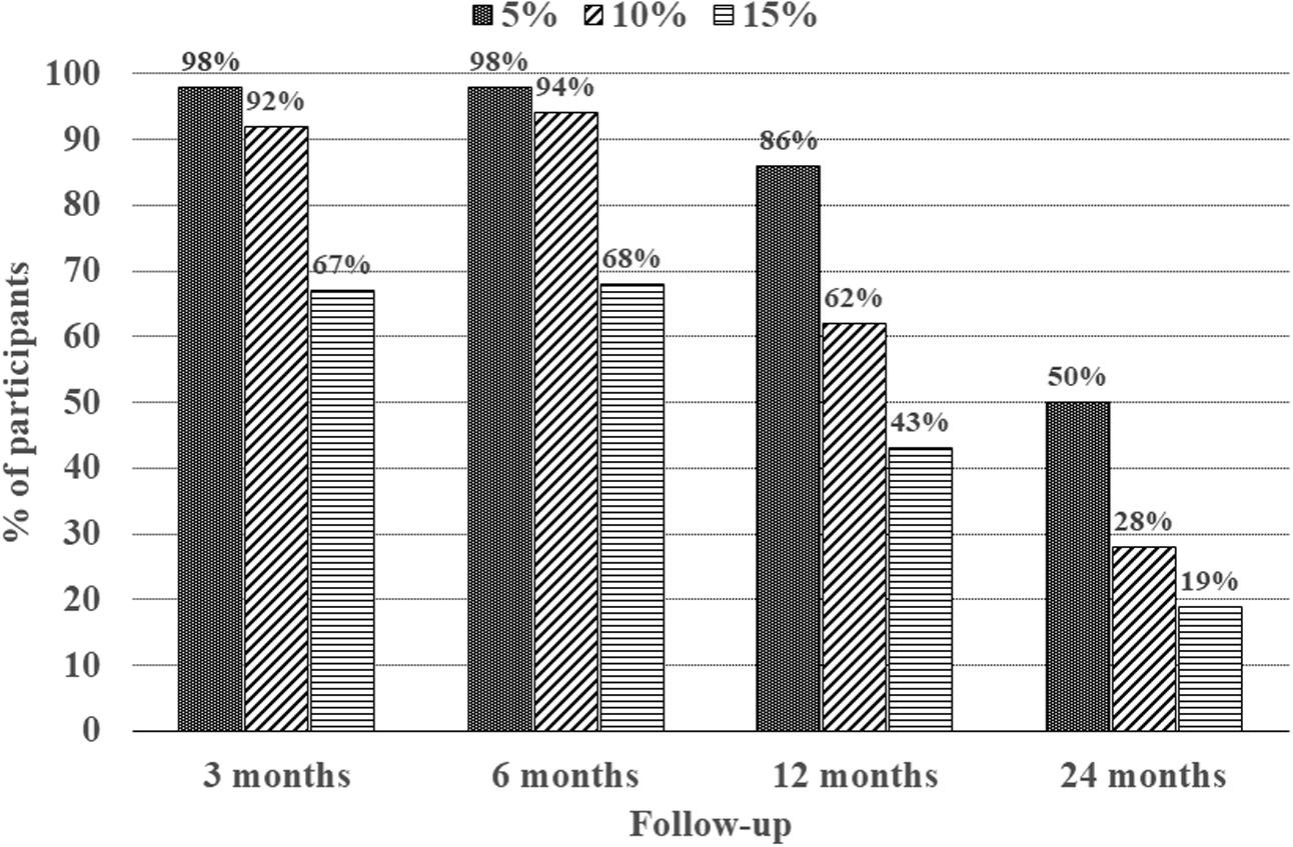
Percentage of participants who met different categorical weight losses (≥5%, ≥10%, and ≥15%) at 3, 6, 12, and 24 months after starting treatment, with follow‐up of 51, 47, 42, and 36 participants, respectively. The weight loss categories are cumulative

Weight change after 3, 6, 12, and 24 months was 16.7% (4.9), 18.9% (6.1), 13.7% (9.4), and 7.2% (10.9), with follow‐up of 51, 47, 42, and 36 participants, respectively (Table 1). The weight loss for women and men was roughly equal. Participants 50 years or older had a weight loss of 17.3% (9.6) after 12 months, compared with 10.5% (7.5) and 12.2% (8.6) for those aged 19–34 and 35–49 years, respectively (data not shown). The proportion who achieved 5%, 10%, and 15% weight reduction after 3, 6, 12, and 24 months is shown in Figure 1.

**TABLE 1:** Mean (SD) BMI and body weight at baseline and at 3, 6, 12, and 24‐month follow‐up

	*n*	BMI	Body weight	Weight change from baseline		SRM	*p*‐value^a^
		kg/m^2^	kg	Kg	%		
Baseline	55	42.0 (6.0)	122.2 (22.9)	–	–	–	–
3 months	51	35.2 (6.0)	102.5 (21.4)	−20.5 (7.3)	16.7 (4.9)	3.41	<0.001
6 months	47	34.2 (5.9)	100.1 (22.7)	−23.1 (8.8)	18.9 (6.1)	3.10	<0.001
12 months	42	36.3 (6.5)	104.3 (23.0)	−16.7 (12.5)	13.7 (9.4)	1.46	<0.001
24 months	36	38.8 (7.4)	113.3 (26.4)	−8.6 (13.2)	7.2 (10.9)	0.66	<0.001

Abbreviations: BMI, body mass index; SRM, standardized response mean.

^a^ Paired sample *t*‐test.

After 12 and 24 months, the attrition rate was 23.6% and 34.5%, respectively. The most common reason for discontinuing treatment was changes in the social situation, for example, a new job, moving to another region, and taking care of children. The highest attrition rate was noted among the youngest participants (19–34 years).

### 3.3 Eating behavior

Changes in eating behavior during treatment showed increased restraint as well as reductions in uncontrolled and emotional eating (Table 2). The effect size (ES) of change in cognitive restraint was large after 6 months (ES = 1.11) and moderate after 12 (0.70) and 24 (0.57) months. The change in uncontrolled eating was small after 6 (0.40) and 12 (0.47) months and moderate after 24 (0.55) months, while the change in emotional eating was small at follow‐up (0.48, 0.36, and 0.47).

**TABLE 2:** Mean (SD) eating behavior scores (TFEQ‐R21) at baseline (*n* = 55) and at 6 (*n* = 43), 12 (*n* = 35), and 24 months (*n* = 22) of follow‐up

TFEQ‐R21	Mean (SD)	SRM	*p‐*value^a^
Cognitive restraint			
Baseline	34.9 (20.2)	–	–
6 months	60.3 (18.5)	1.11	<0.001
12 months	53.8 (17.5)	0.70	<0.001
24 months	55.8 (20.6)	0.57	0.011
Uncontrolled eating			
Baseline	36.8 (21.2)	–	–
6 months	26.9 (20.6)	0.40	0.003
12 months	28.4 (20.6)	0.47	0.005
24 months	27.1 (22.6)	0.55	0.014
Emotional eating			
Baseline	49.1 (28.5)	–	–
6 months	35.9 (30.2)	0.48	0.002
12 months	41.4 (29.2)	0.36	0.060
24 months	39.9 (27.5)	0.47	0.035

*Notes*: Score range 0–100. Higher scores indicate higher levels in the three eating behaviors.

Abbreviations: SRM, standardized response mean; TFEQ‐R21, Three‐Factor Eating Questionnaire‐Revised 21‐item.

^a^ Wilcoxon signed rank test.

### 3.4 Generic HRQoL

**FIGURE 2:**
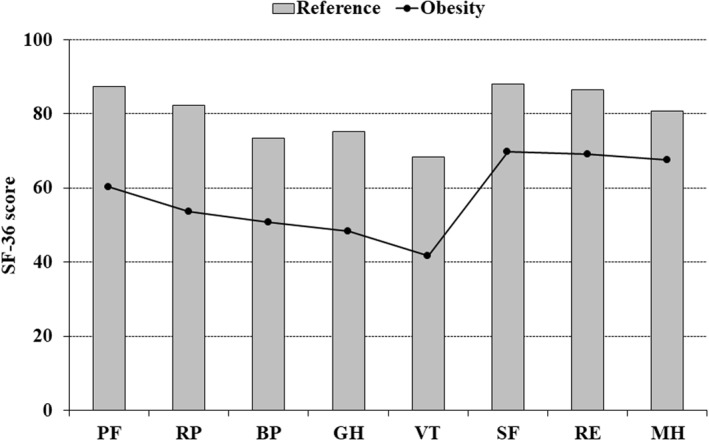
Comparison of the Short Form 36‐item Health Survey (SF‐36) health profile between the study group at baseline, and a gender‐ and age‐matched reference sample (*n* = 715) from the general Swedish population. All comparisons are significant (*p* < 0.001; Mann–Whitney *U*‐test). The scores for the SF‐36 scales range from 0 to 100, and higher scores indicate better health‐related quality of life (HRQoL). PF, physical functioning; RP, role physical; BP, bodily pain; GH, general health; VT, vitality; SF, social functioning; RE, role emotional; MH, mental health; SF‐36, Short Form 36‐item Health Survey. The effect sizes (Cohen's *d*) of the between‐group differences were: PF = 1.41, RP = 0.82, BP = 0.83, GH = 1.19, VT = 1.19, SF = 0.70, RE = 0.49, and MH = 0.63

In Figure 2, the SF‐36 health profile of the study group at baseline was compared with a reference sample from the Swedish general population. All eight subscales had significantly lower scores in the study group and effect sizes indicated large differences on physical functioning (ES = 1.41), role physical (0.82), bodily pain (0.83), general health (1.19), and vitality (1.19), moderate differences on social functioning (0.70) and mental health (0.63), and a small difference on role emotional (0.49).

By the 6‐month follow‐up, the four SF‐36 physical domains (physical functioning, role physical, bodily pain, and general health), as well as vitality and social functioning, had significantly improved compared to baseline (Table 3). The effect sizes of change were large for physical functioning (1.10) and general health (0.98), moderate for role physical (0.67), bodily pain (0.68), and vitality (0.64), and small for social functioning (0.36). No significant changes were seen for role emotional and mental health. After 12 months, significant improvements were observed for physical functioning, bodily pain, general health, vitality, and social functioning. Effect sizes indicated large improvement in physical functioning (1.09) and small improvement for bodily pain (0.40), general health (0.40), vitality (0.37), and social functioning (0.39). At 24 months' follow‐up, physical functioning was significantly improved, with a moderate effect size (0.50), while changes in all other SF‐36 domains were nonsignificant compared to baseline.

**TABLE 3:** Mean (SD) SF‐36 scores at baseline (*n* = 55) and at 6 (*n* = 43), 12 (*n* = 35), and 24 months (*n* = 22) of follow‐up

	PF	RP	BP	GH	VT	SF	RE	MH
Baseline	60.2 (19.6)	53.6 (37.7)	50.7 (27.6)	48.3 (22.1)	41.6 (21.7)	69.7 (30.3)	69.1 (41.0)	67.5 (22.9)
6 months	81.0 (15.9)	82.6 (30.1)	68.1 (28.9)	71.2 (17.9)	60.2 (20.3)	83.4 (20.5)	78.3 (35.5)	73.4 (18.0)
12 months	77.5 (20.7)	59.6 (43.5)	60.3 (29.0)	57.7 (21.5)	50.0 (23.7)	78.6 (23.2)	57.8 (42.9)	68.2 (20.7)
24 months	72.5 (26.7)	53.6 (48.3)	55.0 (32.2)	56.5 (24.4)	53.7 (24.4)	64.8 (33.1)	66.7 (44.7)	67.1 (23.5)
SRM								
6 months	1.10	0.67	0.68	0.98	0.64	0.36	0.17	0.26
12 months	1.09	0.36	0.40	0.40	0.37	0.39	0.20	0.16
24 months	0.50	0.09	0.09	0.21	0.26	0.42	0.05	0.09
*p*‐value^a^								
6 months	<0.001	<0.001	<0.001	<0.001	<0.001	0.040	0.247	0.149
12 months	<0.001	0.050	0.024	0.027	0.019	0.046	0.227	0.522
24 months	0.029	0.749	0.791	0.290	0.095	0.060	0.711	0.945

*Note*: Score range = 0–100, with higher scores indicating better HRQoL.

Abbreviations: BP, bodily pain; GH, general health; MH, mental health; PF, physical functioning; RE, role emotional; RP, role physical; SF, social functioning; SF‐36, Short Form 36‐item Health Survey; SRM, standardized response mean; VT, vitality.

^a^ Wilcoxon signed rank test.

### 3.5 Obesity‐related HRQoL

Based on cutoff values for the OP scale, 31% reported mild psychosocial distress, 29% moderate distress, and 40% severe distress prior to treatment. Distress and avoidance scores significantly improved at 6, 12, and 24 months' follow‐up, with large effect sizes (1.41, 1.03, and 0.93 for distress; 1.13, 0.82, and 0.89 for avoidance; Table 4).

**TABLE 4:** Mean (SD) psychosocial functioning scores (OP), anxiety and depression scores (HAD), and pain severity and interference scores (BPI‐SF) at baseline (*n* = 55) and at 6 (*n* = 43), 12 (*n* = 35), and 24 months (*n* = 22) of follow‐up

	Mean (SD)	SRM	*p‐*value^a^
OP			
Distress			
Baseline	52.4 (24.5)	–	–
6 months	23.4 (17.5)	1.41	<0.001
12 months	29.4 (24.6)	1.03	<0.001
24 months	33.0 (22.7)	0.93	<0.001
Avoidance			
Baseline	36.5 (22.3)	–	–
6 months	15.7 (13.9)	1.13	<0.001
12 months	17.9 (17.4)	0.82	<0.001
24 months	17.6 (17.6)	0.89	<0.001
HAD			
Anxiety			
Baseline	5.9 (4.2)	–	–
6 months	4.5 (3.6)	0.39	0.032
12 months	5.8 (4.1)	0.13	0.347
24 months	6.2 (5.1)	0.08	0.671
Depression			
Baseline	5.7 (4.2)	–	–
6 months	2.8 (2.5)	0.70	<0.001
12 months	4.3 (4.0)	0.31	0.069
24 months	5.5 (4.9)	0.12	0.436
BPI‐SF			
Pain severity			
Baseline	3.1 (2.1)	–	–
6 months	2.3 (2.0)	0.47	0.004
12 months	2.7 (2.1)	0.24	0.158
24 months	3.3 (2.6)	0.11	0.708
Pain interference			
Baseline	3.1 (2.6)	–	–
6 months	1.8 (1.7)	0.71	<0.001
12 months	2.7 (2.5)	0.23	0.038
24 months	3.1 (3.0)	0.07	0.848

*Note*: Higher scores indicate higher dysfunction (OP), more anxiety and depression symptoms (HAD), and worse pain (BPI‐SF).

Abbreviations: BPI‐SF, Brief Pain Inventory‐Short Form (score range 0–10); HAD, Hospital Anxiety and Depression scale (score range 0–21); OP, Obesity‐related Problems scale (score range 0–100); SRM, standardized response mean.

^a^ Wilcoxon signed rank test.

### 3.6 Domain‐related HRQoL

Based on HAD scores at baseline, the proportion of cases with possible and probable anxiety disorder was 20.0% and 14.6%, respectively. A small, significant reduction of anxiety symptoms was observed after 6 months (ES = 0.39), but a return to the baseline value was noted after 12 and 24 months (Table 4).

The HAD scores showed that the proportion of cases at baseline with possible depression disorder was 18.8% and with probable depression disorder, 12.7%. Depression scores decreased significantly to about one‐half at 6 months' follow‐up (ES = 0.70), while no significant change was noted at the 12‐ and 24‐month follow‐up (Table 4).

According to BPI‐SF scores, the proportion at baseline who reported no pain was 18.2%, while 45.5% reported mild pain, 32.7% moderate pain, and 3.6% severe pain. A small improvement in pain severity (ES = 0.47) and moderate improvement in pain interference (ES = 0.71) was observed after 6 months (Table 4). Also, a small improvement in pain interference (0.23) was noted after 12 months, while pain scores after 24 months had returned to baseline levels.

### 3.7 Relationship between attendance at treatment and changes in body weight and HRQoL

The number of treatment visits was significantly associated with greater weight loss: *r* = 0.40 (*p* = 0.006) at 6 months, *r* = 0.48 (*p* = 0.001) at 12 months, and *r* = 0.56 (*p* = 0.000) at 24 months follow‐up, whereas the associations between treatment visits and changes in HRQoL (SF‐36, OP, HAD, and BPI‐SF) were nonsignificant.

### 3.8 Patient‐reported experience measures

After the 3‐month LED phase, 72% reported that it had been easy to follow the LED and 87% stated that they had managed to strictly adhere to the diet regimen. The majority (68%) felt more energetic during the LED, but 19% felt more tired.

Gastrointestinal side effects during the LED were experienced as insignificant by 47%, mild by 40%, and severe by 13%. During the 3 months of gradual phasing out of meal replacements, 76% experienced the side effects as insignificant, 11% as mild, and 13% as severe.

At 3, 6, and 12 months, 98%, 88%, and 85% of participants, respectively, reported that they were fairly to very motivated to continue treatment. Participants were also asked to what extent they were receiving social support from family, friends, workmates, and so on in their efforts to maintain a lower weight. After 3 and 6 months, 96% and 88%, respectively, reported that the support was sufficient. By 12 months, the perceived support had diminished, but 66% thought it was still sufficient.

## 4 DISCUSSION

This study evaluated the effects of a 2‐year behavioral weight loss intervention program in 55 participants with class II or III obesity. The program comprised an initial 3‐month liquid LED (880 kcal/d), followed by 3 months of reintroduction of regular foods. Weight loss after the LED phase was 16.7%, and maximum weight loss after 6 months was 18.9%. Subsequently, significant weight regain was observed and weight reduction in completers after 12 and 24 months was 13.7% and 7.2%, respectively.

The structured and prolonged refeeding phase led to an additional weight loss of about 2% between the 3‐month and the 6‐month follow‐up, which was due to continued negative energy balance after the initial LED phase. Furthermore, as participants were able to focus on reintroduction of one meal at a time for 4 weeks, they had the opportunity to adapt to and practice new dietary habits, which may have had a positive effect on their eating behavior. At 6 months' follow‐up, 94% of the completers had achieved a weight loss of at least 10%. This result confirms previous findings suggesting that food reintroduction after VLED/LED should be structured and slow, occurring over a longer period to enhance weight control.^12^, ^27^, ^28^

Few previous studies have evaluated patient‐reported experiences (acceptability, motivation, adherence, satisfaction with treatment, side effects, social support etc.) of VLED/LED treatment.^29^ In the present study, the majority (72%) of participants thought it was easy to adhere to the LED and most (87%) claimed that they had strictly followed the regimen. In addition, the participants reported high motivation to continue treatment at 3‐ and 6‐month follow‐up, which is probably attributable to the rapid weight loss and support at group meetings. The dropout rate after 6 months was 14.5%, suggesting that the LED and the extended food reintroduction were well accepted by the participants as a strategy to achieve weight loss, which is in line with the conclusions of a review of three qualitative studies.^29^

Only a small proportion of those who succeed in losing weight with nonsurgical methods manage to maintain a clinically relevant weight reduction over the long term.^30^, ^31^ In the present study, 62% and 28% of the completers achieved a weight loss of at least 10% after 12 and 24 months, based on data obtained from 76% and 65% of participants at follow‐up. Attrition from weight loss programs is often due to nonrandom reasons, as participants often do not return for follow‐up if they have poor outcomes. Consequently, high attrition is likely to skew the long‐term results in a more positive direction.

Favorable short‐ and long‐term changes in eating behavior were observed, with an increase in cognitive restraint, and reductions in uncontrolled and emotional eating. This change pattern has been observed in other weight loss studies and is associated with weight reduction as well as healthier dietary habits, such as lower energy and fat intake, higher fiber intake, and less consumption of unhealthy foods.^32^, ^33^, ^34^ Changes in behaviors that improve eating control are among the most consistent determinants of successful weight loss maintenance.^35^ The positive changes in eating behavior are probably a combined effect of behavioral treatment^36^ and the prolonged reintroduction of ordinary foods after LED.

The comparison of the SF‐36 health profile to a gender‐ and age‐matched population sample showed substantially lower scores on all SF‐36 domains in the study group, demonstrating markedly impaired generic HRQoL in individuals with class II and III obesity. A greater negative impact on physical compared to mental HRQoL was seen, which is in line with previous studies.^1^ This also indicates that the margin for improvement in the study group was greater for physical than for mental HRQoL.

After maximum weight loss at 6 months, six of eight SF‐36 domains were significantly improved, with large or moderate effects in five domains. This finding suggests that weight losses of 15%–20% after nonsurgical treatment can have a markedly positive effect on generic HRQoL; however, this result is most likely influenced by the positive experiences during the weight loss phase. Studies of HRQoL outcomes after bariatric surgery show that peak improvements are observed at the end of the weight loss phase. Thereafter, gradual weight regain, as well as deterioration of HRQoL, is seen up to 6 years after surgery.^37^, ^38^ At 12 months' follow‐up in the present study, improvements in generic HRQoL had declined compared to the levels at 6 months, although large improvement in physical functioning and small effects in four other domains were still observed. Therefore, while a weight loss of 10%–15% after a period of successive weight regain may result in improvements in physical HRQoL, especially physical functioning, the effects on mental HRQoL are minor. At 24 months, only physical functioning was moderately improved (12 scale points), which can be considered a clinically significant effect. This demonstrates that 5%–10% weight loss may improve physical functioning, but no other generic HRQoL domain, which is in line with several other studies.^1^ However, improving physical functioning in persons with obesity is an important treatment goal, especially as it can allow the individual to more readily engage in physical activity.

The OP scale measures the impact of obesity on psychosocial functioning, a key domain in the assessment of HRQoL in people with obesity.^21^ Because obesity is a stigmatized condition, individuals with obesity can develop disturbances in psychosocial functioning.^21^, ^39^, ^40^ Participants in the present study reported high levels of weight‐related psychosocial distress prior to treatment, with a mean OP distress score of 52.4, which is slightly lower than the pre‐treatment score of 61.6 observed in surgical candidates in the Scandinavian Obesity Surgery Register.^41^ Reducing weight stigma was one of the goals of the intervention program in the present study. Major improvements in OP distress and avoidance scores were noted at the follow‐ups, both in the short and in the longer term, which is probably due to a combined effect of weight loss and behavioral treatment. A few previous studies have shown that behavioral programs can improve psychosocial functioning despite only modest weight loss,^42^, ^43^ suggesting that behavioral treatment may have an independent beneficial effect on weight stigma and obesity‐specific HRQoL. This is an important finding because internalized weight stigma among persons with obesity has been associated with adverse physical and mental health consequences, as well as maladaptive behaviors such as unhealthy eating and avoidance of physical activity,^40^, ^44^ which may negatively interfere with weight loss outcomes.^39^ Therefore, reducing body weight and weight stigma can be complementary goals in behavioral programs. By focusing on reducing internalized stigma, beneficial effects on general health and HRQoL may be achieved, in addition to the effects of weight reduction.^39^, ^45^ However, further research is needed to test the potential weight loss‐independent effects of behavioral treatment on weight stigma in controlled studies.

In our study, HAD anxiety and depression scores were significantly reduced at 6 months' follow‐up, suggesting a positive treatment effect on mental wellbeing. The improvement was most pronounced for depression symptoms, with a moderate effect size. Previous research indicates that depression symptoms may decrease after longer VLED treatment and accompanying substantial weight loss, especially in combination with behavioral therapy and prescription of low intensity physical exercise.^46^ Ein et al. found no effect of VLED on anxiety symptoms, although few of the studies they reviewed had assessed anxiety symptoms after VLED. In the present study, no improvements in anxiety and depression were observed after 12 and 24 months, suggesting that greater weight loss may be required to achieve effects on mood. However, as even the beneficial effects of bariatric surgery on mental health are small,^3^ it is possible that weight loss intervention alone is insufficient to improve mental HRQoL in the long term, among individuals with severe obesity.

The short‐term improvements in HAD scores in the present study show that the instrument is more sensitive in detecting changes in mental wellbeing compared to the SF‐36 mental health scale, which did not show any statistically significant improvement after 6 months. Therefore, to detect changes in mental wellbeing following weight loss treatment, domain‐specific instruments should be used.

Obesity is associated with various types of chronic pain conditions,^47^ especially musculoskeletal pain,^48^ which interferes with daily functioning and may have a considerable negative impact on HRQoL. Weight loss can relieve symptoms and pain, and the effects of bariatric surgery show that large proportions of patients experience improvements in bodily pain and functioning after surgery.^49^ In the present study, the BPI‐SF was used to assess pain and an advantage of the instrument is that it provides separate scores for pain severity and pain interference. The mean pain intensity score at baseline was about 3 points, indicating mild pain on average in the study sample, although only one‐fifth of the participants reported no pain prior to treatment. Both the pain intensity and the pain interference scores improved after 6 months, demonstrating that a mean weight loss of 18.9% can reduce symptoms and pain, as well as negative impacts of pain on daily activities, function, and wellbeing. However, pain scores after 24 months had returned to baseline levels, indicating that 5%–10% weight loss is insufficient to reduce pain among persons with severe obesity.

Developing strategies to maintain weight loss is the overall goal of all nonsurgical obesity treatments. Programs that combine VLED/LED or behavioral treatment with pharmacotherapy may result in greater weight loss compared to monotherapy.^5^, ^50^ Pharmacotherapy was not used in the present study, but may be an adjunct to the treatment program to improve long‐term outcomes.^28^ However, in Sweden, only one of the three approved anti‐obesity drugs is subsidized by the state, which limits its use.

A major limitation of this study is the lack of a control group. Many studies, however, show that weight reduction in control groups with minimal support is about 1% after 1 year,^5^ indicating that placebo effects during weight loss treatment are negligible, mainly due to the powerful environmental, physiological, and behavioral barriers to weight loss.^30^, ^31^ Another limitation of the study is the poor follow‐up of patient‐reported outcomes after 24 months, which indicates that these data should be interpreted with some caution. Furthermore, information on the participants' physical activity is lacking and increased activity levels are considered to be an essential factor for long‐term weight loss maintenance.^51^

## 5 CONCLUSION

In persons with class II and III obesity, a 3‐month liquid LED followed by 3 months of food reintroduction, in combination with behavioral group treatment, was associated with a substantial weight loss of 18.9% after 6 months. Favorable changes in eating behavior were observed as well as extensive short‐term improvements in HRQoL, comprising the physical, psychosocial, and mental domains. At 12 months, weight loss was 13.7% and improvements in HRQoL had declined compared to the 6‐month levels. Positive effects were mainly observed for physical and obesity‐specific HRQoL. After 24 months, a weight loss of 7.2% was associated with a moderate improvement in physical functioning and a large improvement in psychosocial functioning. The effect on psychosocial functioning is most likely related to both weight loss and behavioral treatment.

## CONFLICT OF INTEREST

The authors declare that they have no conflict of interest to disclose.

## AUTHOR CONTRIBUTIONS

Jan Karlsson conceived the study and its design. Jan Karlsson and Marije Galavazi led the analysis and writing. Johan Jendle and Stefan Jansson took part in the analyses, as well as critically reviewing the manuscript. All authors have approved the final version of the article.
